# Supplementary figures and images for: Genetic stock structure of the silky shark Carcharhinus falciformis in the Indo-Pacific Ocean
Source: PLoS One. 2023 Oct 12;18(10):e0292743. doi: 10.1371/journal.pone.0292743 (PMC10569576; doi:10.1371/journal.pone.0292743)

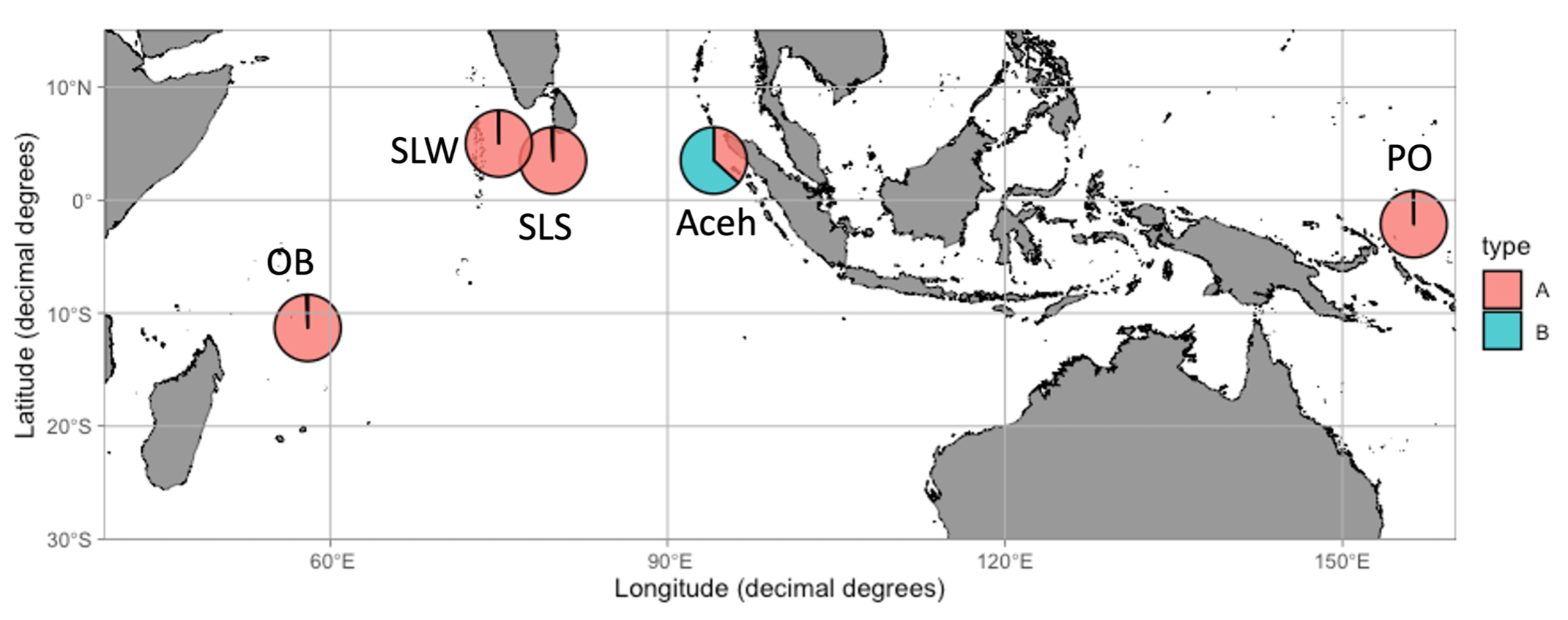

Supplement: S1 Fig — (PNG) [file pone.0292743.s007.png]
