# Supplementary material for: Genetic stock structure of the silky shark Carcharhinus falciformis in the Indo-Pacific Ocean
Source: PLoS One. 2023 Oct 12;18(10):e0292743. doi: 10.1371/journal.pone.0292743 (PMC10569576; doi:10.1371/journal.pone.0292743)
Supplement: Attachment — Submitted filename: Responses to Reviewers0727.docx [file pone.0292743.s008.docx]

Responses to Editor's comments:

Add details (table) on the sampling procedure and samples used.

The introduction seems a little too long and broad for the content of the paper, shorten please.

We are concerned about the effective population size estimate, since sample from one populations was very small.

The authors are urged to significantly improve the language and style of the manuscript, which currently contains many linguistic, stylistic, and grammatical issues.

**R: We have rearranged and shorten the introduction and rests of the comments raised by the reviewers have been properly addressed, the revision is also polished by English editing company.**

Journal requirements:

**1. The adjustments have been made including the file's name and the manuscript.**

**2. The role of the funder in the study has been added to the cover letter”** **Additionally, the funders had no role in study design, data collection and analysis, decision to publish, or preparation of the manuscript.”**

**3. Figure 1, 3 and 4 were generated using ggOceanMap and ggplot2 packages and the citations of these two packages have been added. ggOceanMaps uses openly available geographic data and the source of the map (Natural Earth data) was also acknowledged.**

Reviewer #1

The introduction seems a little too long and broad for the content of the paper. The authors should also spend some time on improving the flow of this section. At the moment there are several abrupt topical changes between individual paragraphs, which make it difficult for the reader to follow the overall story.

**R: We have rearranged and rephrased the paragraphs to make it more concise.**

Line 43-45: Please use updated numbers, reference 3 is outdated.

**R: The numbers have been updated.**

Line 55-57: There are a lot more oceanic shark species than the ones mentioned, so better replace “i.e.” with “e.g.”

**R: The correction has been made.**

Line 110-111: Kraft et al. 2020 is missing in the reference list.

**R: The citation and reference have been added.**

Please include a description of how the Monmonier’s algorithm was used in your analysis as results for this method are presented in the results section but there is no mention in the methods section.

**R: We have added a description as follows “The barrier analysis was applied to detect the genetic boundary between the population based on Monmonier's (1973) algorithm (53) function in Adgenet v1.3.4 package (51) in R which detects boundaries among geographic populations with both geographic and genetic distances as valuated graph. This is achieved by finding the path exhibiting the largest distances between connected populations.” in the methods section.**

Line 131-140: More details on sample collection need to be included, e.g. how were sampled sharks identified, what kind of tissue samples were collected, which part of the body was sampled, were samples collected from the whole animal, etc.

**R: More details on sample collections have been added as follows “A total of 307 tissue samples were collected from 5 locations, including the Southwestern Pacific Ocean (PO, N = 116), Indonesia Aceh fish market (Aceh, N = 41), Sri Lanka Negombo and Colombo fish market (SLW, N = 68), Mirissa and Kudawella fishing ports (SLS, N = 40), and Western Indian Ocean (OB, N = 42) (Figure 1) from September 2018 to January 2021. A small tissue of fin or muscle (1 cm^3^) was collected from each shark and preserved immediately in 95% EtOH.”**

Middle initials are missing for most authors in the reference list and must be included before publication, e.g. 41, 62, 64 and many more.

& In-text citations are sometimes in name-year style, sometimes in number style.

**R: The correction has been made.**

Reviewer #2

I have a small concern regarding the effective population size data, since one of the populations has a very small sample size that can be affecting your final results. The statistical analyses is rigorous and appropriate, again only one minor concern regarding the effective population size results.

&

Line 239
Be careful with these samples sizes! I think they could be underestimating Ne in Aceh.

**R: I understand your concern and we had the same concern as well. In order to exclude this potential effect we ran a separate test by randomly reduce the sample size of Indian Ocean and Pacific Ocean to 41 and the results showed a similar figure as we got in the Table 4. Which indicate that the estimation of the Ne based on the genetic variations in Aceh population were referring by its potential low genetic diversity (isolated population).**

Line 316-317 I see higher genetic diversity in Aceh, do you think this has to do with a higher number of samples from this location?

Also, the results of very low Ne from this location do not make sense with the genetic diversity found.

&

Line 339-342 You should check bias in estimates of Ne when samples sizes are low:
This is an article you should check out:
Estimating effective population size from linkage disequilibrium: severe bias in small samples Phillip R. England1,*, Jean-Marie Cornuet2 , Pierre Berthier3 , David A. Tallmon4 & Gordon Luikart5

**R: Thank you for your valuable comment, the sample size of Aceh is the second lowest among 5 populations, therefore, it unlikely the higher Ho was due to higher number of samples in this population. However, we admit that during the pooling process the overall heterozygosity may change accordingly then could ultimately influence the Ne estimation. Therefore, we added a paragraph to address these potential influences and added the reference suggested by reviewer as follows “Although serval factors may affect the Ne that estimated in the present study, including the smaller sample size of Aceh (N=41) used may cause a potential underestimation of the Ne (78) and the potential bias caused by pooling samples from different geographic locations (Indian Ocean population). The Ne that we estimated in the present study may not represent the exact Ne; instead, it is a relative Ne value estimated based on the genetic variations in each population, which indicates that the silky shark inhabiting the Aceh waters could be an isolated and endemic population.”**

Line 66-67 Review this sentence, you refer to the silky shark as plural and then as singular.

**R: The corrections have been made.**

Line 72 Sometimes you refer to the silky shark as “the” silky shark and sometimes not, I suggest you are consistent throughout the paper.

**R: The corrections have been made.**

Line 75 Sometimes you refer to the silky shark as “the” silky shark and sometimes not, I suggest you are consistent throughout the paper.

**R: The corrections have been made.**

Line 103 The genetic population structure

**R: The correction has been made.**

Line 108 "if" the focal species instead of "while" the focal species

**R: The correction has been made.**

Line 109-111 Review this sentence

**R: We rephrased it as “Nuclear markers are commonly used to discriminate the genetic structure of sharks (ie. Blackmouth catshark, 38), making them a valuable tool in stock identification studies.**

Line 110 This article is not on the reference list, do you refer to this article : Genomics versus mtDNA for resolving stock structure in the silky shark (Carcharhinus falciformis)

**R: The reference has been added.**

Line 146 Samples were amplified? I suggest you write down what were the reagents and their concentrations for the PCR reaction.

**R: We have added more info as follows “Each reaction contained 50ng DNA, 12.5µL TaqDNA polymerase 2X master Mix RED (0.4 mM each dNTPs, 15 mM MgCl, 0.2 unit of Ampliqon DNA polymerase) (Ampliqon Denmark) and each of primers (200nM). Genotyping was carried out by ABI sequencer.”.**

Line 149 PCR not PRC

**R: The corrections have been made.**

Line 160 I suggest exact test in addition to Fst

R: We have revised the sentence as “Pairwise F_ST_ value with Fisher’s exact test was **estimated using MSA 4.05”**

Line 193 You do not present the results of HW and linkage disequilibrium for each locus

**R: We have add the results of HWE and LD in S2 and S3 Table, respectively and added a paragraph to described the results as “The results of HWE test showed that most of the populations at locus Cafa16 and Cafa44 were deviated from HWE, and significant LD mainly occurred in Aceh across all loci (S2-S3 Table).”**

Line 201 Sampling locations plural

**R: The correction has been made.**

Line 232 Figure 4 I suggest you describe this barriers in the Figure heading to better understand the image.

**R: We added the following sentence in the Figure caption as “The thickness of blue lines represented the levels of the effect of barriers on gene flow.”**

Line 296 Are there any other examples of this same pattern of genetic structure with other marine organisms?

**R: So far, there were only few cases which focus on giant clam and seagrass showed population from Aceh were genetically different others by using microsatellite loci, but as for pelagic species our case seems to be the first one. Since the driving force for the genetic partitioning of those demersal species may be different from the pelagic species, therefore, we did not include these literatures in the discussion part.**

Line 355 Sphyrna lewini not Sphyrna lewinin

**R: The correction has been made.**
