# Supplementary material for: Genetic stock structure of the silky shark Carcharhinus falciformis in the Indo-Pacific Ocean
Source: PLoS One. 2023 Oct 12;18(10):e0292743. doi: 10.1371/journal.pone.0292743 (PMC10569576; doi:10.1371/journal.pone.0292743)
Supplement: Attachment — Submitted filename: Responses to Reviewers.docx [file pone.0292743.s009.docx]

Responses to Editor's comments:

Reviewer 2 points out LD in Aceh sampling site may affect the estimation of various population genetic statistics. At first glance, this may be caused by mixture of ancestry of shark in that location. The effects of this could be tackled by subdivision of the Aceh sample and reestimation of statistics. Please discuss and respond.

**R: We agree that the LD found in Aceh could possibly be due to the mixing of two genetic stocks, which aligns with what we mentioned in our discussion. However, if we separate Aceh into two sub-populations the sample size of each will be too small to perform further population genetic analyses. Therefore, in response to Reviewer 2’s comment and concerns specifically on the potential effect of loci Cafa16 and Cafa44 which deviated from HW. We excluded Cafa16 and Cafa44 and rerun Fst, Structure and Ne estimation, the results were similar to those based on 8 loci, except that the Ne value derived from 6 loci is smaller than that derived from 8loci for the PO population (Table S5). This suggests that adding these two loci didn't affect our observed patterns. Even if we separated these two subpopulations (cluster I and II), the results of LD test showed that cluster I from Aceh showed significant LD still for all the comparisons. Considering the small Ne we found in Aceh, this LD may be caused by genetic drift under a small population size as observed in the human population ( Laan and Pääbo, 1997).**

**Reference:**

**Laan, M., & Pääbo, S. (1997). Demographic history and linkage disequilibrium in human populations. Nature genetics, 17(4), 435-438.**

The language still requires more work. Reviewer1 provides amble editing suggestions, but many others can also be offered. Best if a third party reads this over and helps with grammar and clarity of text.

**R: We appreciate the feedback and highlighting the concerns regarding the language issues in our manuscript. We have sent the revised manuscript for language editing again to improve the quality of this latest revision.**

Reviewer #1

Despite the authors employing an “English editing company”, concerns regarding language issues remain. These issue need to be fixed throughout the document, before the manuscript is ready for publication. I have just listed a few examples below to illustrate my concerns. Most mistakes are minor and can easily be fixed.

**R: We sincerely appreciate the reviewers’ comments regarding the language issue.**

**In this revision, we have corrected the issues mentioned by Reviewer 1 and then sent the revision to an English editing company for a comprehensive language editing service before submitting it back to PlosOne. We hope this version meets the publishing standard of PlosOne.**

Line 19: replace “for ” with “in”

**R: The correction has been made.**

Line 20-22: Here, we examined 307 individuals of silky sharks from 5 sampling locations across Indo-Pacific to reveal their genetic stock structure and effective population size by using eight microsatellite loci.
Should read:
Here, we used eight microsatellite loci to examine the genetic stock structure and effective population size of 307 silky sharks across 5 Indo-Pacific sampling locations.

**R: The correction has been made.**

Line 28: “We suggest ”

**R: The correction has been made.**

Line 39-42: Check language and grammar - Elasmobranchs' k-selected life history characteristics (slow growth, late maturity, low fecundity, and long lifespan) have made them more vulnerable to overexploitation and have less potential for recovery than r-selected bony fish (1, 2).

**R: The sentence has been revised to : Due to their K-selected life history traits, including slow growth, late maturity, low fecundity and long lifespan, elasmobranchs are more susceptible to overfishing and have lower recovery potential compared to r-selected bony fish (1, 2)**

Line 42-45: Check language and grammar - High global demands for shark products like fins, skins, gill plates, liver oil, and jaws has led to over 391 elasmobranch species being categorized as threatened by the International Union for Conservation of Nature (IUCN) (3).

**R: The sentence has been revised to: High global demand for shark products, such as fins, skins, gill plates, liver oil, and jaws, has led to over 391 elasmobranch species being categorized as threatened by the International Union for Conservation of Nature (IUCN) (3)**

Line 45-49: Check language and grammar - Numerous shark species have also been listed in the Convention on International Trade in Endangered Species of Wild Fauna and Flora (CITES) Appendix I and Appendix II, which restricted their international trade due to decreasing populations during the past few decades (4, 5). This situation urgently needs comprehensive management strategies for wild populations to reach the goal of future sustainable use.

**R: These sentences have been revised to : Numerous shark species have also been listed in Appendix I and Appendix II of the Convention on International Trade in Endangered Species of Wild Fauna and Flora (CITES), restricting their international trade in response to declining populations over the past few decades(4, 5). This situation underscores the urgent need for comprehensive management strategies to ensure the future sustainable use of wild population.**

Line 54: “patterns”

**R: The correction has been made.**

Line 55 Here and throughout the document: Please check the use of scientific and common names. As a general rule: At first mention, both the scientific and the common name, as well as the authority are given. After first mention you can choose whether to use the common or scientific name throughout the remainder of the document, but you must be consistent.

**R: The correction has been made. Scientific and common names were provided for each species in the first mention. Subsequently, we used the common names throughout the manuscript.**

Line 61: sex-biased

**R: the correction has been made.**

Line 66-67: “In pelagic and coastal regions”

**R: the correction has been made.**

Line 73-76: Check language and grammar - “It is one of the most exploited shark species and the most common bycatch species of the tuna longline and purse seine fishery due to their overlapping representation with schools of tuna (16, 24, 25), their fins with high demand and abundant in the trade markets of Guangzhou, Hong Kong, and Taiwan (26-29).

**R: These sentences have been revised to : It is among the most exploited shark species and is commonly caught as bycatch in tuna longline and purse seine fishery due to its overlapping presence with tuna school (16, 24, 25). Additionally, there is a high demand for its fins, which are abundantly traded in markets in Guangzhou, Hong Kong, and Taiwan (26-29).**

Line 77-78: Check language and grammar - To date, the silky shark has been listed as a ‘Vulnerable’ species on IUCN Red List (30) and Appendix II species on the CITES to control the international trade of this species.

**R: These sentences have been revised to :To date, the silky shark has been listed as a ‘Vulnerable’ species on IUCN Red List (30) and as an Appendix II species under CITES to regulate its international trade.**

Line 96: add space

**R: The correction has been made.**

Line 107: Add “silky shark” before “population”

**R: The correction has been made.**

Line 115-116: Samples used in the present study are fish landing which were no longer alive when we obtained the tissue sample for population genetic analysis.

**R: The sentence has been revised to : Samples used in this study were from fish landings; the specimens were no longer alive when we collected tissue samples for population genetic analysis.**

Line 258: “Karft” should read “Kraft”

**R: The correction has been made.**

Reviewer #2

I have one concern:

The results of HWE test showed that most of the populations at locus Cafa16 and Cafa44 were deviated from HWE, and significant LD mainly occurred in Aceh across all loci (S2-S3 Table). This is concerning. Do you think the LD is affecting your results? No loci with LD should be included in the analyses!! These results were not included in the initial submission that is why I had not addressed them earlier.

Statistical methods for analyzing HWE, FSTs, Ne ect.. assume independence between loci, so the presence of strong LD can violate this assumption and potentially lead to biased results!!

**R: We appreciate the thoughtful feedback provided by the reviewer and have taken steps to address these concerns to ensure the robustness of our findings. To assess the potential impact of these two loci, we conducted an analysis using STRUCTURE, MSA, and Neestimator with the dataset excluded these two loci to examine the population structure patterns and the effective population size.**

**Our analysis indicated that the exclusion of Cafa16 and Cafa44 did not substantially alter our results.**

1. **STRUCTURE analysis: The bar plot generated from the STRUCTURE analysis exhibited a pattern consistent with the original analysis, suggesting that the population structure remain unchanged.**
2. **Pairwise F_ST_ analysis: The results of the pairwise F_ST_ analysis were aligned with the patterns based on 8 loci.**
3. **Effective population size: In the case of effective population size estimation, when employing the reduced data of 6 loci (excluding Cafa16 and Cafa44), we did observe a decrease in population size for the PO population (617.7 to 142.9). However, the other two populations exhibited a similar effective population sizes based on 8 loci.**

**As we provided the results of those estimations in the supplementary material which could exclude the potential effect due to deviated from HW on Cafa16 and Cafa44, therefore, we would like to keep the original tables to provide a better resolution regarding the identification of genetic stock.**

**In addition to the above replies, we have discussed the mechanisms may drive the results of LD in the section of effective population size as follow:**

**This is further supported by the deviation from LD for all loci in the Aceh population, which could be influenced by several factors, including selection, genetic drift, inbreeding, demographic history and population subdivision (79). However, this situation is unlikely to be caused by natural selection and inbreeding in our case since the inbreeding coefficient of Aceh was the lowest (FIS= 0.02), suggesting that it could be a population with random mating. Theoretically, individuals in small and isolated populations tend to inbreed (80). Nevertheless, in some sharks (e.g., the sicklefin lemon shark Negaprion acutidens and the basking shark), specific mating behaviors have evolved to avoid inbreeding during the mating season (81, 82) Therefore, we suggest that the silky shark in Aceh may also exhibit special mating behavior or migratory patterns to reduce the chance of mating with close kin. However, further research on mating behavior is needed to provide additional evidence supporting this hypothesis. The potential effect by mixture of ancestry of the silky shark in Aceh population has also been evaluated by separating Aceh population into two sub-populations based on cluster I or cluster II they belong to (Fig 3) and reran the LD test, the results still showed a significant linkage for all comparisons of the cluster I sub-population (S6 Table). Therefore, the most likely explanation for the LD we observed is the small population size of Aceh. In small populations such as Aceh population in our case, the effect of genetic drift tends to be stronger than in larger populations, and the demographic fluctuation (i.e. population bottleneck) could also act as a potential mechanism to cause the findings we observed.**
